# Healthcare professionals’ experiences of being observed regarding hygiene routines: the Hawthorne effect in vascular surgery

**DOI:** 10.1186/s12879-021-06097-5

**Published:** 2021-05-04

**Authors:** Francis Rezk, Margaretha Stenmarker, Stefan Acosta, Karoline Johansson, Malin Bengnér, Håkan Åstrand, Ann-Christine Andersson

**Affiliations:** 1 Department of Clinical Sciences, Malmö, Lund University, Malmö, Sweden; 2 Department of Biomedical and Clinical Sciences, Linköping University, Linköping, Sweden; 3 Unit of Vascular Surgery, Department of Surgery, Region Jönköping County, Jönköping, Sweden; 4 Department of Clinical and Experimental Medicine, Linköping University, Linköping, Sweden; 5 Department of Paediatrics, Institute of Clinical Sciences, the Sahlgrenska Academy at the University of Gothenburg, Gothenburg, Sweden; 6 Department of Health, Medicine and Caring, Linköping University, Linköping, Sweden; 7 Jönköping Academy, Jönköping University, Jönköping, Sweden

**Keywords:** Healthcare professionals, Compliance, Adherence to standard precautions, Hygiene observation, Hawthorne effect, Hierarchy, Vascular surgery

## Abstract

**Background:**

The Hawthorne Effect is the change in behaviour by subjects due to their awareness of being observed and is evident in both research and clinical settings as a result of various forms of observation. When the Hawthorne effect exists, it is short-lived, and likely leads to increased productivity, compliance, or adherence to standard protocols. This study is a qualitative component of an ongoing multicentre study, examining the role of Incisional Negative Pressure Wound Therapy after vascular surgery (INVIPS Trial). Here we examine the factors that influence hygiene and the role of the Hawthorne effect on the adherence of healthcare professionals to standard hygiene precautions.

**Methods:**

This is a qualitative interview study, investigating how healthcare professionals perceive the observation regarding hygiene routines and their compliance with them. Seven semi-structured focus group interviews were conducted, each interview included a different staff category and one individual interview with a nurse from the Department for Communicable Disease Control. Additionally, a structured questionnaire interview was performed with environmental services staff. The results were analysed based on the inductive qualitative content analysis approach.

**Results:**

The analysis revealed four themes and 12 subthemes. Communication and hindering hierarchy were found to be crucial. Healthcare professionals sought more personal and direct feedback. All participants believed that there were routines that should be adhered to but did not know where to find information on them. Staff in the operating theatre were most meticulous in adhering to standard hygiene precautions. The need to give observers a clear mandate and support their work was identified. The staff had different opinions concerning the patient’s awareness of the importance of hygiene following surgery. The INVIPS Trial had mediated the Hawthorne effect.

**Conclusion:**

The results of this study indicate that the themes identified, encompassing communication, behaviour, rules and routines, and work environment, influence the adherence of healthcare professionals to standard precautions to a considerable extent of which many factors could be mediated by a Hawthorne effect. It is important that managers within the healthcare system put into place an improved and sustainable hygiene care to reduce the rate of surgical site infections after vascular surgery**.**

**Supplementary Information:**

The online version contains supplementary material available at 10.1186/s12879-021-06097-5.

## Introduction

Surgical site infections (SSIs) continue to be of major concern for both patients and the healthcare system, and can jeopardise the results of vascular surgery [1], leading to increased length of hospital stay and costs, and higher rates of readmission, amputation and mortality [1, 2]. SSIs are among the most common healthcare-associated infections (HAIs) [3]. Prevention of these infections is complex and requires the integration of a range of preventive actions and measures before, during, and after surgery. To reduce SSIs and maintain low infection rates [4, 5], bundle of care approaches have proven to be important [4, 5], such as improved hygiene routines, and perhaps shifts of antibiotic prophylaxis therapy [6, 7]. WHO has also developed Global guidelines on the prevention of surgical site infection to provide a comprehensive range of evidence-based recommendations for interventions to be applied during the pre-, intra- and postoperative periods for the prevention of SSI [8]. Open vascular surgery in the lower extremities is associated with a high risk of SSIs, where such measures have been reported to have no retained effect [6]. Therefore a multi-centre randomized controlled trial investigating the effect of negative pressure wound therapy (NPWT) on closed incisions was warranted [9]. Many units in the present study centre are engaged in the care vascular surgery patients, some of them follow a checklist but some do not. Therefore, it is important to examine if the ongoing INVIPS-Trial, observations, and such checklist could mediate, i.e., imply a Hawthorne Effect, (HE).

## Background

The World Health Organization launched the global hand hygiene programme in 2009 to reduce HAIs and improve patient safety. Evaluation and feedback on hand hygiene performance is not only important elements of this programme but they are one of the consensus recommendations of its guidelines [10]. Hand hygiene could be improved when healthcare professionals (HCPs) know that they are under observation, however, such observation has some potential bias. These changes in behaviour are often attributed to the well-known HE [11]. The HE is a type of observer effect, and is often cited as a source of bias in observed behavioural changes among study participants, or due to infection control interventions [12, 13]. Although the HE is frequently mentioned in the scientific literature, there is considerable inconsistency concerning the description and definition of the phenomenon. The most important and consistent concept of the HE is a change in behaviour due to the participants awareness of being observed [14]. The change in behaviour occurs after participants become aware of being observed, and the size and direction of the change in behaviour depend on the total time the participant is aware of being observed [15]. The HE is a non-specific treatment effect; it is a change in behaviour as a motivational response to the interest, care, or attention received through observation and assessment. The HE also has a performance ceiling and the performance impact decreases with continued observation past peak performance [15]. It is not clear how HE affects human behaviour [12] or how HCPs think and express in what way their behaviour and ways of working change when being observed. The correlation between improved compliance with hand hygiene routines and a reduction in the rate of HAIs has been well documented [16]. To obtain a sustainable and constant Hawthorne effect associated with improved compliance with hand hygiene routines, decreased infection and cross-transmission rates could certainly represent an ideal perspective [17]. Increased adherence to standard precautions, mediated via the HE, would thus probably reduce the rate of SSIs after vascular surgery particularly under ongoing prospective randomized INVIPS-trial at the present study centre.

### Local context

The Department for Communicable Disease Control (DCDC) at the Jönköping County Hospital has overall responsibility for hygiene routines and guidelines intended to prevent and reduce the risk of infections within the healthcare system in the county of Jönköping, Sweden. The basic requirements are adherence to standard precautions, protocols, and the use of protective clothing. There are nominated hygiene observers at each healthcare unit where patients are examined, treated, or cared for. The director of each unit appoints a hygiene observer, and the DCDC provides them regular training twice a year. According to local recommendations, each unit is expected to monitor adherence to standard precautions by carrying out direct observations of about 20% of all employees each month. Furthermore, HCPs are encouraged to regularly rate their perceived adherence to these hygiene observers using a simple self-reporting protocol, (see Additional file 1), created for this purpose [18]. This protocol is based on WHO Hand Hygiene Technical Reference Manual [19] and SOSFS 2015:10, National Board of Health and Welfare regulations on basic hygiene in health care [20]. This procedure has been in place since 2006, although compliance measurements have only been mandatory since 2009. The present study centre has a high documented rate of SSI following vascular surgery (> 40%), and to reduce this, it has reverted back to a previous antibiotic prophylaxis regimen [7]. As it is important to understand why the SSI rate is so high [7], research collaboration was initiated with Lund University. Surgeons at the University Hospital in Lund/Malmö have a long-standing interest in NPWT, in both open and closed wounds. It has been shown that incisional NPWT has the potential to reduce infection rates [21–23]. The present study centre is one of four centres in an ongoing multi-centre randomized controlled trial (registered at Clinical Trials. gov, identifier: NCT0191313) comparing closed incisional NPWT with standard dressings for the prevention of SSI after vascular surgery (INVIPS Trial) [24]. It is highly likely that the HCPs involved in this randomized controlled trial will experience a HE [9, 24]**.**

### Aims

The aim of this study was to examine how HCPs perceive being observed when following hygiene routines, and how they believe and express how these observations affect their way of working, and thus their adherence to standard precautions.

## Methods

### Design, setting and participants

**Fig. 1:**
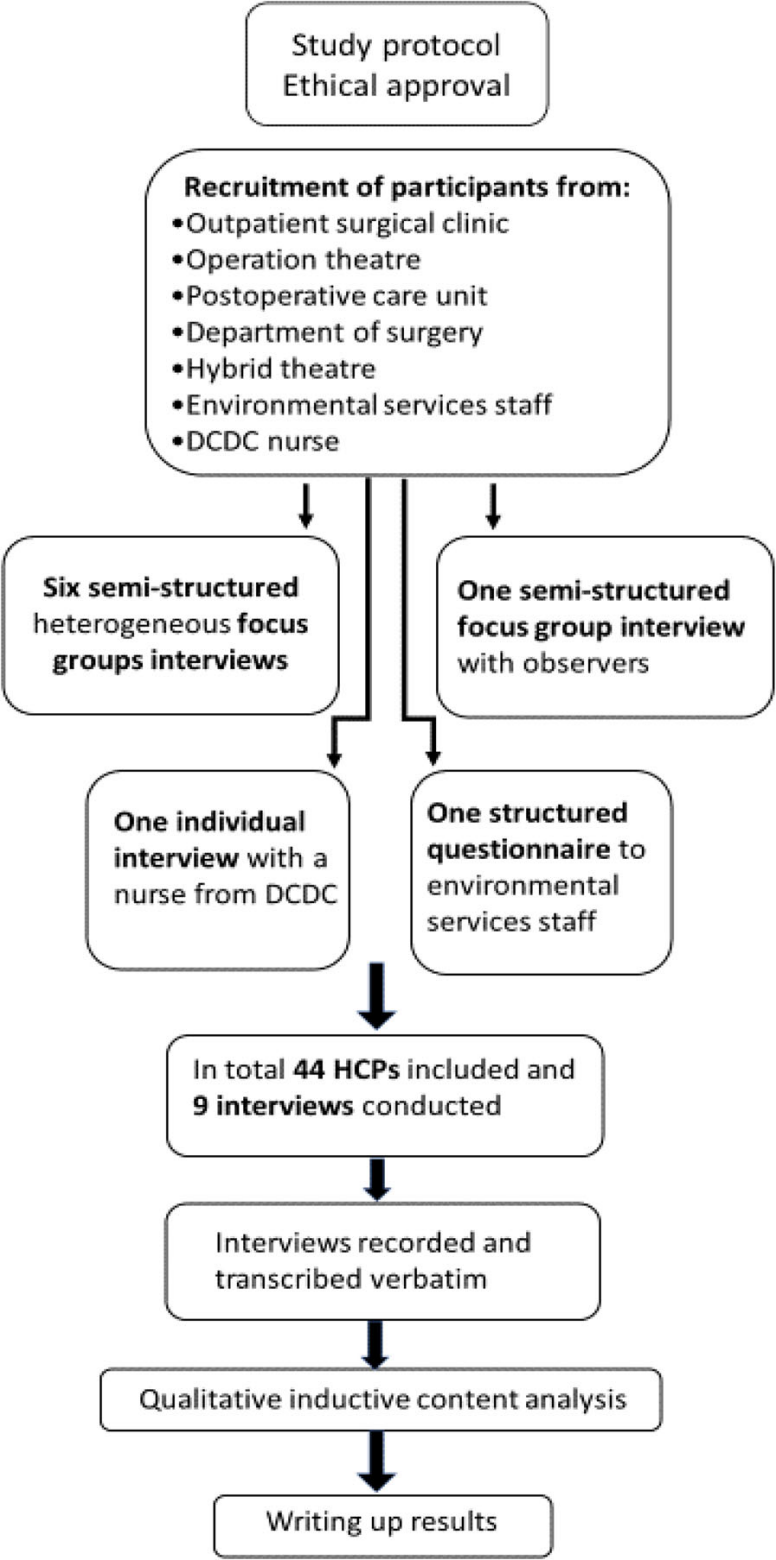
The organisation of the study

The study is an explorative qualitative case study, Fig. 1. Before starting the study, several information meetings were organized with the staff, senior surgeons, and unit managers from all the clinics and units involved.

### Participants

The staff at the relevant units were invited to participate using a convenience sample. The staff who were on duty on the days of the focus group interviews were asked to take part by the unit manager or the nurse responsible for the physician’s schedule. Staff who did not want to participate in the interviews, were able to decline without implications. None of participants volunteered without being asked. Multi-professional focus groups of 44 HCPs were formed, consisting of 19 nurses, 15 assistant nurses, five observers and five of a total of seven vascular surgeons at the present study centre (the remaining two vascular surgeons are the researcher and one of the co-authors). The researcher was not involved in inviting or interviewing of the participants because he works as a vascular surgeon and moves routinely between all study recruitment areas. The experience of the participants in healthcare work varied from two to more than 10 years as outlined in Table 1, (see Additional file 2). The study included HCPs engaged in vascular surgical patient care and who had been observed by the local hygiene observers, in the pre-, peri- and postoperative care at the Department of Surgery. Nurses and assistant nurses were recruited from outpatient clinics, the operation theatre, the Postoperative Care Unit, the Department of Surgery and the DCDC. Environmental services staff (ESS) were also included in the study. The HCPs invited to participate were given written and oral information about the study and provided written informed consent to be interviewed. The participants also received information about the reasons for joining the INVIPS-trial, mainly the high frequency of SSI.

**Table 1:** the numbers of the participants and their experience

Number of participants	Years of experience
5	1–2 years
20	5–10 years
19	More than 10 years

### Interviews and data collection

Seven focus group interviews on seven different occasions were conducted with heterogeneous groups of nurses, assistant nurses and vascular surgeons being observed, using a semi-structured questionnaire guide. Separate interview was carried out on observers (one nurse and four assistant nurses), an individual interview of a nurse from the DCDC, while ESS filled out a structured questionnaire interview, (see Additional file 3), after having rejected to be part of the focus groups interviews. The interviews took place at the study center in an enclosed room. An interview guide, (see Additional file 4) was used, which started with the all-encompassing introduction: *“The focus group interview is about your experiences of, and how you perceive that observations of hygiene routines affect your work”.* Questions were asked regarding the factors that were most likely to have had an impact on compliance, such as feedback, self-assessment, antibiotics, hand hygiene, introduction of new HCPs, education, adherence, wound care, and collaboration. The participants were generally active and engaged in the discussions. The author moderating the interviews and the authors acting as observers were not employed in any of the participating units. The first interview served as a pilot, although included in the analysis, and was performed by the moderator alone, since the assistants were occupied with healthcare work. The HCPs were interviewed as part of the ongoing study, Incisional Negative Pressure Wound Therapy after vascular surgery (INVIPS Trial) [9], between October 2019 and January 2020. The focus group interviews lasted between 67 and 90 min and were recorded and transcribed verbatim. The interview with the nurse from the DCDC lasted 35 min. Communication with the ESS were carried out by answering target questions via an e-mail, as they did not wish to take part in an oral interview. Sex interviews sessions were performed, although no new relevant data emerged after the fourth interview session.

### Data analysis

The findings of the interviews were analysed based on the qualitative inductive content analysis approach described by Elo and Kyngäs [25]. This method of analysis is usually applied when new areas are studied, or when a known area is to be reviewed from a new perspective [25]. Data analysis included open coding, general categorization and main categorization [25]. Prominent statements were then highlighted as meaning units, and open codes created by making notes in different organized tables. The codes were then collected on coding sheets and grouped as general categories. Finally, the main categories were charted by comparing and contrasting the general categories. Data coding was carried out by two of the authors independently, and then compared and discussed. In cases of disagreement over coding, the codes were discussed, and the original transcript checked, until a consensus was achieved.

### Rigour and trustworthiness

The criteria outlined by Schwandt et al. were used to ensure the trustworthiness of the research [26]. The interviews were conducted successively in a well-defined period during the ongoing INVIPS Trial [9]. The data were analysed independently, using an inductive content analysis approach [25] to achieve dependability. Credibility was also ensured through field notes, memos and reflections on the purpose and main research questions. Peer checking and member checking techniques were used by asking all the authors to review the analysis process and the results.

## Results

**Fig. 2:**
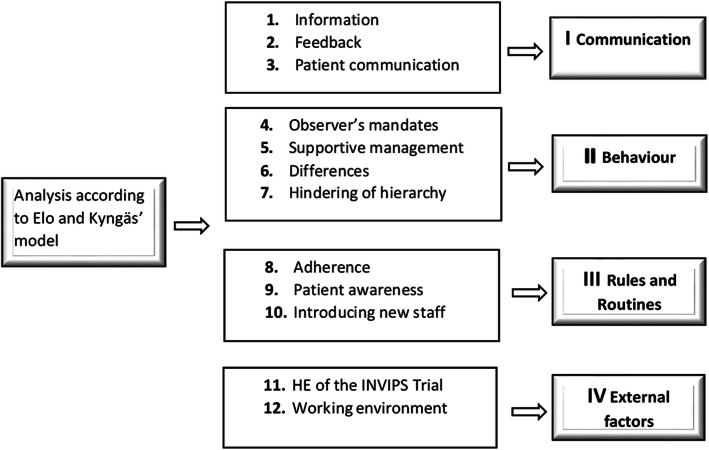
The four main themes and 12 subthemes identified from the analysis according to qualitative inductive content analysis as described by Elo and Kyngäs [25]. HE = Hawthorne effect

The analysis of the interviews revealed four main themes and 12 subthemes (Fig. 2). HCPs mentioned in the interviews that their perception of being observed was affected by many factors. The complexity and factors are explained in the contents of the four themes and are illustrated by quotations.

### The four main themes and 12 subthemes identified

#### The importance of communication

Communication was found to be crucial, and this theme consisted of the subthemes Information, Feedback, and Patient communication. Most of the participants complained about communication especially that between assistant nurses and other HCPs. They emphasized the importance of group meetings between all units engaged in the care of vascular surgery patients, in order to share knowledge and experiences. The participants also expressed a desire for more individual direct feedback rather than on group level. Another important factor was the lack of an open friendly climate that allowed everyone to speak up when someone made a mistake. According to the participants, regular updates, feedback, reminding each other, and easy access to information on all hygiene routines improved compliance. As the ESS did not carry out observations, they had no way of expressing their opinions to the DCDC. They did not think there was any need for observations. According to the ESS, there were considerable differences in cleaning routines and duration between different units. These were especially noticeable between the operating theatre and the other units. A lack of communication was reported between HCPs and ESS concerning the clinical condition of inpatients, particularly concerning those with infections or recently operated patients.

*“We don’t know why a patient is on a ward but as a rule, we don’t have to go into the room and clean if staff are treating the patient. We don’t know what they are doing.”*

#### Behaviour influences

The second theme, Behaviour, consisted of the subthemes Differences, Hierarchy, Observer’s mandates, and Supportive management.

Considerable differences were found in terms of specific roles, the degree of hierarchy and the use of checklists at the various units. The HCPs perceived that hygiene routines were better in the surgical department than in other units, but were poorest in the outpatient clinic due to the heavy workload and the variety of patients and treatments. The observers reported a high degree of hierarchy; for example, vascular surgeons seldom reacted to comments by assistant nurses on adherence to standard precautions, or their responses were sometimes uncooperative or even patronizing.

*“That's why it's so hard, sometimes when you point something out, that a person is sloppy, some of them get upset and moody when you tell them, and then, yes but we just do it.”*

Some participants said that some HCPs invented their own hygiene routines based on questionable personal beliefs. They also reported a lack of observations and hygiene awareness among medical students, visiting physicians, and particularly HCPs in the anaesthesiology team in the operating theatre.

*“On the anaesthesia side, for example, that it is dirty and clean, … they, the surgeon, can run around to the hospital entrance and then go into an operating room wearing the same shoes, … then it’s not acceptable to go in with the wrong cap, but that’s also a bit of ‘making up your own rules’.”*

The study revealed that most observers were assistant nurses. This is sometimes seen as a problem, as they do not have sufficient seniority, and occasionally feel that they do not have the support of management. They feel it is difficult to give advice as they are in the lowest hierarchical position. In all units except the operating theatre, the observer worked alone. They also suggested a stronger network, ideally initiated, and run by the DCDC so that all the observers could meet, at least once a year, to exchange experiences. The observers also expressed a desire for more support from the DCDC such as meetings and joint activities. They also thought that there should be more than one observer in each unit, then they could support each other.

*“ … and in the unit, it’s the assistant nurse who has a specific interest in this, I think. And then perhaps you should clarify their roles, surgical assistant nurses are so well established, while assistant nurses in the other departments are not as well established.”*

Nurses working at the DCDC also claimed that the only way to maintain good, long-term adherence to standard precautions is for all HCPs to become aware of the importance of observations. However, they pointed out that there is a need for continuous efforts to achieve this goal. HCPs expressed their concern about the possible relation between mobile phones and SSI, particularly in the operating room. They said that there were always several phones in the operating room during surgical procedures.

#### Adherence to rules and routines

Rules and routines emerged as the third theme, and consisted of Adherence, Patient awareness, and Introducing new staff. Most of the HCPs emphasized the importance of observations in the prevention of contamination and infections. HCPs are aware that they do not always follow the correct procedure, and do not use the self-assessment, system sufficiently. All the participants knew that certain routines should be followed, but they did not always know where to find information on them. This was not only a problem for the individual, but also when introducing new members of staff. They lacked easy access to relevant digital information on adherence to standard precautions and the results of observations. Many suggestions for improvements were discussed, including the installation of a terminal providing easy access to such information. Hygiene requirements are most rigorous in the operating theatre and are therefore usually followed, but even here, some HCPs applied their own routines. The participants had different opinions regarding the patient’s awareness of the importance of hygiene following surgery. Both vascular surgeons and highly experienced HCPs stated that observations had no impact on their contact with patients, and that they worked as usual. The study revealed a lack of patient participation in their own postoperative hygiene, which may be due to a lack of information or misunderstanding. Different opinions were expressed among the HCPs as to whether the patients were given such information, and how well they understood it and followed it.

Many factors affect the efficiency of the introduction and training of new HCPs. The most frequent problems are a shortage of time, lack of information, and inadequate communication. The introduction of new HCPs was perceived to increase the workload of existing HCPs. Vascular surgeons reported a lack of training in hygiene and adherence to standard precautions after completing their training.

#### External factors

The fourth theme identified was External factors, in the form of the ongoing Incisional Vascular Surgical Wound Protection by Negative Incisional Wound Therapy (INVIPS-Trial), and Work environment. The participants stated that the INVIPS-Trial had improved their adherence to standard precautions through modification of their behaviour. However, the vascular surgeons and highly experienced HCPs stated that the INVIPS-Trial had no impact on their treatment of patients. They reported that they acted in the same way regardless of whether the patient was part of the study or not; and that observations were of greater importance.

The above findings show that the participants’ adherence to standard precautions could be significantly influenced by working environment factors. Working at maximum capacity, a shortage of time, multi-patient rooms, staffing shortages, and the unavailability of equipment could have negative effects. High workload may increase the frequency of non-compliance to standard precautions.

*“… we have so few single rooms, thereby, few private toilets to every patient … We mix patients, there are various patients with infectious diseases, we try to give them separate rooms, however, they come in and may be having throat boils or any infection , so it's just that you heard the manger discuss what reasons to this patient variations and so on about everything else and not about its risks”*

The observers had no dedicated time to make observations, which had to be carried out when the opportunity arose. Many factors beyond their control, such as shortages due to sickness, sometimes led to observations not being carried out. The observers sometimes had to record their observations after working hours or during their lunch breaks.

*“Resources, it's a bit difficult to talk about that on the ward, today we had five HCPs, some days we have none. Therefore, I do it only when I have time. When I have a little time over I usually sit and write or register the observations data after work or when I take my lunch break. It would be better if we had proper time for such things … there are always problems, I think they need more staff.”*

The participants expressed concern that other patients were admitted to the same surgical ward as vascular surgery patients. Otolaryngology, ophthalmology, maxillofacial and endocrine surgery patients shared rooms at the units. Having patients with different diagnoses led to greater rotation of staff between the different teams at the unit, which led to the feeling they were providing poorer care to the vascular surgery patients. This may cause stress among HCPs, thus reducing their adherence to standard precautions.

*“... so we always explain it to the new HCPs during their introduction, but of course, we have different patients in our department and so maybe you rotate and forget about it afterwards, you may not be there in that team for a month. I belong to the vascular team, ... but tomorrow I can have ear patients.”*

## Discussion

The hospital where this study was conducted, has a well-developed organization for the management of issues related to education and training in hygiene, observation processes, improvements, and the HCPs adherence to standard precautions*.* The findings of this study revealed that compliance is affected by many factors, not least a lack of communication between different groups of HCPs. Many HCPs maintained that communication was vital in the care of vascular surgery patients. Most of the HCPs, especially assistant nurses, stressed the importance of verbal reporting on patients specifically about the postoperative care of surgical wounds and the prescription of antibiotics. Inadequate communication and a hierarchical arrangement of healthcare providers foster hostility, frustration and distrust, which hinder collaboration and jeopardize the quality of patient care [27, 28]. Lack of use of self-assessment by HCPs is an important issue to resolve in the hospital, as self-assessment is one of the most essential factors in preventing HAIs in patients [29]. The need to improve the observation process and the lack of use of self-assessment reinforce the importance of communication currently available in WHO tools, especially WHO Hand Hygiene Technical Reference Manual [19].

Constructive and regular feedback is extremely important in ensuring long-term compliance, which in turn will lead to a reduction in nosocomial infections and SSIs. Lewis et al. concluded that an audit and feedback system may be an effective means of improving the quality of care and reducing practice variability within a surgical department [30]. Furthermore, they showed that the number of SSIs and readmissions were significantly reduced in the high-acuity procedures in head and neck surgery after the feedback period, compared between two assessment periods, the pre- and post-feedback periods [30]. They also suggested that it was possible that the performance of the surgical staff improved, through the HE, as they were aware that they were being audited [30]. The communication between HCPs and vascular surgery patients was not clear regarding the perioperative perception of information on the operation. This indicates the need for better communication between HCPs and their patients to increase the patient’s awareness of the need for self-care after surgery and during healing. Such an interaction could strongly influence the patient’s understanding of their condition, and their attitude to self-care [31], possibly reducing the frequency of SSIs after vascular surgery [32]. A separate qualitative study on the interaction between HCPs and vascular surgery patients is warranted.

The findings of this study indicate that direct observations are generally effective, but that observation has a smaller effect on the most experienced HCPs and vascular surgeons. The HCPs expressed the importance of direct observation, not only by the observers but also by reminding each other. If the DCDC were to cease hygiene observations, then hygiene-related problems at the units would probably increase, apart from in the operating theatre, in where it was felt that there was already an open climate allowing constructive feedback. The overall interpretation of the findings was that the direct observation method was perceived positively among HCPs. On the other hand, they were dissatisfied with the lack of feedback from management, observers, and from each other. The observers pointed out that they needed the support of management and the DCDC. Management must hold HCPs accountable and give the observers a mandate. A lack of support to observers can reduce the effectiveness of interdisciplinary communication and collaboration [33], resulting in poor compliance among the most experienced HCPs in vascular surgery patient care. Supporting HCPs generally benefits patient outcomes and may thus also reduce SSIs. Therefore, we suggest that observers be given greater support, including a clear mandate and higher status. The findings of the present study confirm those of Reeves et al., that confused roles, effects of professional socialization, and power and status differentials hinder interprofessional collaboration [34].

Hierarchy was identified as a major problem, particularly differences in status between assistant nurses and physicians. A hierarchical structure is a major obstacle to cooperation, which may lead to poor compliance and thus jeopardize patient safety. To improve the situation, it is necessary to address the current hierarchical professional structure inherent in the healthcare system [33]**.** Lancaster et al. concluded that, *“A hospital patient care model based on the conductor-less orchestra model would mitigate hierarchy; recognize physician, nurse, and unlicensed assistive personnel’s contributions to care; promote improved communication and collaboration; and enhance patient safety.”* [33].

The differences in compliance between the various categories of HCPs were related to the position they held. Vascular surgeons were not included in hygiene instruction, possibly because it was assumed that this was not necessary. However, they could also benefit from such training. Physicians not only exhibited poor compliance, but they also sometimes expressed erroneous beliefs. This finding is in line with that of a hand hygiene compliance study, in which it was found that nurses’ compliance in hand hygiene was better than that of physicians [35]. Similarly, Erasmus et al. found that nurses’ compliance was higher than that of doctors and other healthcare workers in 25 of 44 studies on the association between profession and hand hygiene compliance [36]. Continuous training and the improvement of professional skills among the medical staff regarding hospital hygiene are necessary to reduce HAIs, mainly SSIs.

The HCPs at the operations theatre demonstrated a high level of compliance as a result of their open climate with less hierarchy, better teamwork and the use of checklists. However, they highlighted the poor compliance of the anaesthesiology team. The use of checklists in the operating theatre could have mediated a HE , leading to better compliance. They perceived and experienced that the checklist improved their behaviour and adherence to hygiene precautions, when being observed. Haynes et al. found that the use of a checklist led to changes in both systems and in the behaviour of individual surgical teams. They also found that the implementation of the checklist was associated with concomitant reductions in the rates of death and complications and that the overall rates of SSI and unscheduled reoperation also declined significantly [37].

The participants in this study voiced their concern regarding contamination by mobile phones. Numerous studies have mentioned possible bacterial contamination from mobile phones, although there is no evidence of a direct association between the environmental pathogens on mobile phones and the rate of HAIs [38]. Further studies are needed to clarify the question of whether the use of mobile phones by HCPs constitutes a risk to the patient.

The attitude to introducing new HCPs was positive. However, this was negatively affected by external factors such as high workload and lack of time among the staff. In agreement with Knoll et al. [39], we found that the compliance of HCPs could be significantly negatively affected by external factors such as high workloads (especially in connection with a lack of human resources), which HCPs perceived as disturbing and stressful. Therefore, improving the working environment could lead to better adherence to standard precautions.

The ongoing INVIPS Trial was found to be an external factor that increased awareness among staff and should thus lead to higher compliance with hygiene routines and adherence to standard precautions. The trial has alerted staff to the high postoperative infection frequency at the study centre and made them aware of the importance of hygiene, especially in postoperative wound care. This increased awareness could mediate a HE, but to different degrees among HCPs. The HE would probably have been lower among vascular surgeons and staff with long experience [40].

We would like to emphasize the importance of including ESS, and their role in the hospital’s environmental high-touch surface cleaning, which is an important component of a multifaceted infection control strategy to prevent HAIs [41]. The written answers given by the ESS revealed that there was a lack of communication concerning the status of inpatients, particularly those who had recently undergone surgery, which may influence the risk of contamination. Yanke et al. [42] stated that the ESS may represent an underappreciated resource for hospital infection prevention, and further efforts should be made to engage ESS as members of the health care team. Further efforts should be made to engage these “invisible staff” as part of the healthcare team and culture of infection prevention [42].

The present study implies that improved basic preventive measures have a central role in reducing bacterial transmission and development of SSI. Indeed, in a recent randomized trial, Loftus et al. [43] found that improved basic perioperative preventive measures reduced transmission and SSI by *Staphylococcus aureus*, perhaps the most common pathogen in the hospital setting. The successful seven-component bundle of care in the perioperative setting included efforts in hand hygiene, vascular access care environmental cleaning, organization of the anaesthesia work area quarterly feedback, targeted ultraviolet C light therapy (Helios) in operating environments that had been exposed to *Staphylococcus aureus* and for patient decolonization [43].

### Practical implications

1. Easy access to hygiene routines, hygiene education for all HCPs regardless of role. Information, feedback, and results. The hospital has now started to use an electronic tablet providing easy access to these routines and information via direct links.
2. All HCPs shall be required to follow the hospital’s SHPs. This means filling in self-assessment protocols, and not following personal hygiene routines.
3. Multidisciplinary buy-in is essential to changing the culture of acceptance of feedback from any observer to any HCP.
4. Anaesthesiologists and ESS should be included in the observation process.
5. The implementation of checklists for the various tasks involved in patient care.

## Conclusions

All the staff participating in this study considered that observations of how well hygiene routines are followed are important. To ensure better adherence to standard precautions, the observers must have better backup from managers and the DCDC. It is necessary to establish systematic professional training and education of HCPs concerning hygiene, and to continuously monitor and evaluate the level of compliance in clinical practice, particularly in vascular surgery. Compliance among HCPs can also be improved by regular training and feedback, improving communication, interprofessional educations, and training opportunities can be a way to break down the hierarchical structures and communication. ESS should be included in the observation process and communication with them should be improved. Good compliance was mediated through the HE in most of the HCPs, nevertheless, physicians and highly experienced staff were less frequently influenced by the HE. High levels of adherence to standard precautions by all HCPs could reduce the SSI rate after open vascular surgery in the lower extremities.

### Areas for improvement and limitations of the study

The lack of an open friendly climate that allowed everyone to mention mistakes, occasional insufficient seniority of the observer and lack of support from management were identified areas for improvement in order to legitimate the observers mandate and need for change in cultural behaviour. The researcher, a vascular surgeon, noted that the observers rarely observed surgeons while they washed and sterilized their hands before surgery. Therefore, the surgeon’s behaviour and attitude towards this was not monitored. The ESS does not make observations in the present study centre, and they have therefore not any possibility of expressing their opinions to the DCDC. It is acknowledged that their input in the interviews would have been valuable, but they declined to participate, which is a limitation of the study. Higher external validity of the findings would have been achieved in a multicentre qualitative study.

## Supplementary Information

**Additional file 1.** Structured Questionnaire for Environmental services staff.

**Additional file 2.** Self-reporting protocol for participants’ self-assessment of adherence to basic hygiene precautions and dress routines.

**Additional file 3.** Demographic Questionnaire including background characteristics as about profession, experiences, and workplace.

**Additional file 4.** The study Interview guide.

## Data Availability

Due to confidentiality the raw data (i.e., transcribed interviews) will not be made public, although details concerning the analysis process can be provided upon request.

## Abbreviations

DCDC: Department for Communicable Disease Control *at* Jönköping County Hospital
ESS: Environmental services staff
HAIs: Healthcare- associated infections
HCPs: Health care professionals
HE: Hawthorne Effect
INVIPS: Incisional negative pressure wound therapy for the prevention of surgical site infection
NPWT: Negative pressure wound therapy
SSI: Surgical site infection
WHO: World Health Organization
