# Supplementary material for: Healthcare professionals’ experiences of being observed regarding hygiene routines: the Hawthorne effect in vascular surgery
Source: BMC Infect Dis. 2021 May 4;21:420. doi: 10.1186/s12879-021-06097-5 (PMC8097954; doi:10.1186/s12879-021-06097-5)
Supplement: Supplementary file 1 — Additional file 1. Structured Questionnaire for Environmental services staff. [file 12879_2021_6097_MOESM1_ESM.docx]

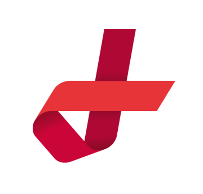

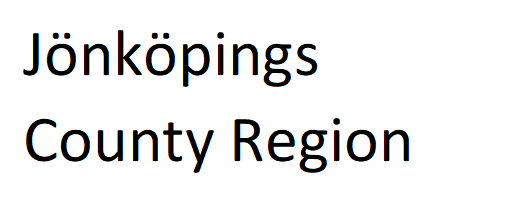
 **Supplementary; Self-reporting protocol**

**PROPERLY DRESSED AND CLEAN HANDS**

**- self-assessment** 2018-04-11

| **Information** | | | | |
| --- | --- | --- | --- | --- |
| The form is used for self- assessment of adherence to basic hygiene precautions and dress routines. Self-assessment is made only by healthcare professionals who have had close contact with caretakers in nursing, examination, or treatment. The form is handed out unprepared, for example under the coffee break, work meeting, or another meeting. Choose a patient and work situation you have experienced today. Ex the first patient you were with today. Fill in the form and leave it to your hygiene representative directly, who will make a summary. | | | | |
|  | | | | |
| Date | Profession | | | |
| Specify the type of nursing, examination, or treatment | | | | |
| **Resultat** | | | | |
| Used an alcohol-based hand rub immediately before working close to the patient |  | Yes | No |  |
| Used an alcohol-based hand rub immediately after working close to the patient |  | Yes | No |  |
| Appropriate use of gloves (see below) during patient care |  | Yes | No | Not applicable |
| Appropriate use of disposable plastic apron (see below) during patient care | | Yes | No | Not applicable |
| Use of short-sleeved work clothes |  | Yes | No |  |
| Without ring, bracelet, and watch |  | Yes | No |  |
| Without different support rails, plasters |  | Yes | No |  |
| Short nails, without nail polish and artificial material |  | Yes | No |  |
| Short or hair up |  | Yes | No |  |
| Comment | | | | |

**Correct basic hygiene precautions**

- **Hand Hygiene with Alcohol-Based Formulation** means that the hands are rubbed directly **before** and immediately **after** close work to patients and before and after the use of gloves.
- **The use of gloves means** that gloves are used in case of risk of contact with bodily fluids or if there is a risk of heavy contamination of the hands. Gloves are changed between each patient and between different care moments of the same patient
- **The use of disposable plastic apron means** to protect the work clothes if it is at risk of being splashed or contaminated with bodily fluids or other biological material, such as in contact with undressed patient.

**Correct working rules and clothes**

- **Clothing means** short-sleeved work clothes that do not go beyond the elbow.
- **Hands** and forearms should be without rings, watch, bracelets, and various support rails in order to be able to do a proper hand hygiene.
- **Nails** are short and free from nail polish or artificial material.
- **Length of hair means** that the hair which is longer than shoulder length, and risks hanging down, should be set up in such a way that it does not hang down in the working field.

**Department for Communicable Disease Control at Jönköping County Hospital**
