# Supplementary material for: Healthcare professionals’ experiences of being observed regarding hygiene routines: the Hawthorne effect in vascular surgery
Source: BMC Infect Dis. 2021 May 4;21:420. doi: 10.1186/s12879-021-06097-5 (PMC8097954; doi:10.1186/s12879-021-06097-5)
Supplement: Supplementary file 2 — Additional file 2. Self-reporting protocol for participants’ self-assessment of adherence to basic hygiene precautions and dress routines. [file 12879_2021_6097_MOESM2_ESM.docx]

**Supplementary; Demographic Questionnaire**

**Healthcare professionals´ experiences of being observed regarding hygiene routines during a randomized trial on vascular surgical site infections**

A study of health care professionals' experience and compliance with hygiene practices __________________________________________________________________________________

**Participants in focus group interview**

**Profession:**

- Nurse
- Assistant Nurse
- Vascular Surgeon

**Experience of working in vascular surgery:**

- Three months-maximum one year
- More than one year - maximum of five years
- More than five years - maximum ten years
- More than 10 years

**Workplace:**

Fill in one or more options where you work/have worked at The County Hospital

- Hospital Ward
- Outpatient surgery reception
- Operating theatre
- Post-operative care unit
- Hybrid operating theatre
