# Supplementary material for: Healthcare professionals’ experiences of being observed regarding hygiene routines: the Hawthorne effect in vascular surgery
Source: BMC Infect Dis. 2021 May 4;21:420. doi: 10.1186/s12879-021-06097-5 (PMC8097954; doi:10.1186/s12879-021-06097-5)
Supplement: Supplementary file 3 — Additional file 3. Demographic Questionnaire including background characteristics as about profession, experiences, and workplace. [file 12879_2021_6097_MOESM3_ESM.docx]

**Supplementary; Structured Interview guide**

**Healthcare professionals´ experiences of being observed regarding hygiene routines during a randomized trial on vascular surgical site infections.**

**Structured Questionnaire for Environmental services staff**

Do you as environmental services staff have any form of hygiene education and/or training?

- If yes, what does the education contain, and what does it comprehend?
- Do you have written routines or memos to follow?
- How are new staff introduced regarding hygiene routines and memos?
- Are there hygiene observers within your organization who conduct hygiene observations and report to the Department for Communicable Disease Control?
  - If so, do you get feedback from those observations, and how do you get that feedback?
  - If not, do you think it would be useful for you to be observed?
- Does cleaning and hygiene routines differ between different departments, such as operation theatre, postoperative care unit, outpatient surgical clinic, and department of surgery? - How in your opinion?
- What do you think about the strengths and weaknesses of hygiene education? Do you want to update and improve this training? - How? This may be difficult to answer if there are no education in place, but what would you like to have?
- Is there communication and collaboration with healthcare staff at the units regarding hygiene issues? If so, in what way? When does such cooperation take place? Would there need to be more communication?
- Is there anything special you are aware of when cleaning staff comes in contact with patients who recently have had surgery?
- Feel free to add more comments and thoughts about your role as environmental services staff regarding hygiene and how to improve your role in the hygiene process. ……………………. ……………………………………………………………………………………………………………………………………………….

Thank you for your time!
