# Supplementary material for: Healthcare professionals’ experiences of being observed regarding hygiene routines: the Hawthorne effect in vascular surgery
Source: BMC Infect Dis. 2021 May 4;21:420. doi: 10.1186/s12879-021-06097-5 (PMC8097954; doi:10.1186/s12879-021-06097-5)
Supplement: Supplementary file 4 — Additional file 4. The study Interview guide. [file 12879_2021_6097_MOESM4_ESM.docx]

**Supplementary; Study Interview guide**

**Healthcare professionals´ experiences of being observed regarding hygiene routines during a randomized trial on vascular surgical site infections.**

**Interview guide**

**Introduction:**

As stated in the information, this focus group interview is about your experiences and perceptions of being observed and if and how this affect work, in relation to the INVIPS-Trial.

**Themes that should be covered in the interview:**

Education and introduction to adherence to standard precautions

Hygiene observations – how it works, usefulness, affection

Adherence to standard precautions (hand hygiene, clothing) – routines and compliance, how ensure?

Self-report and hygiene protocol for self-reporting – use and usefulness?

Follow-up and feedback results – how it works and how/if used?

**Study-specific questions**

Before surgery – instructions for staff, importance of hygiene for patients – checklists, observations in operation theatre

During surgery - observations in operation theatre, the specific study population

After-care – wound dressings (differences in study populations and other patients), antibiotics and other treatments,

Patient self-care – information and follow-up

**General reflections:**

Something else you want to share – other experiences related to hygiene

Thanks for participating!
